# Schizophrenia and cardiometabolic abnormalities: A Mendelian randomization study

**DOI:** 10.3389/fgene.2023.1150458

**Published:** 2023-04-06

**Authors:** Noushin Saadullah Khani, Marius Cotic, Baihan Wang, Rosemary Abidoph, Georgina Mills, Alvin Richards-Belle, Benjamin I. Perry, Golam M. Khandaker, Elvira Bramon

**Affiliations:** ^1^ Division of Psychiatry, Mental Health Neuroscience Department, University College London, London, United Kingdom; ^2^ Department of Genetics and Genomic Medicine, UCL Great Ormond Street Institute of Child Health, University College London, London, United Kingdom; ^3^ Camden and Islington NHS Foundation Trust, London, United Kingdom; ^4^ Division of Psychiatry, Epidemiology and Applied Clinical Research Department, University College London, London, United Kingdom; ^5^ Department of Psychiatry, University of Cambridge, Cambridge, United Kingdom; ^6^ Cambridgeshire and Peterborough NHS Foundation Trust, Cambridge, United Kingdom; ^7^ MRC Integrative Epidemiology Unit, Population Health Sciences, Bristol Medical School, University of Bristol, Bristol, United Kingdom; ^8^ NIHR Bristol Biomedical Research Centre, Bristol, United Kingdom; ^9^ Avon and Wiltshire Mental Health Partnership NHS Trust, Bristol, United Kingdom

**Keywords:** schizophrenia, Mendelian randomization, single-nucleotide polymorphism, cardiometabolic traits, metabolic syndrome

## Abstract

**Background:** Individuals with a diagnosis of schizophrenia are known to be at high risk of premature mortality due to poor physical health, especially cardiovascular disease, diabetes, and obesity. The reasons for these physical health outcomes within this patient population are complex. Despite well-documented cardiometabolic adverse effects of certain antipsychotic drugs and lifestyle factors, schizophrenia may have an independent effect.

**Aims:** To investigate if there is evidence that schizophrenia is causally related to cardiometabolic traits (blood lipids, anthropometric traits, glycaemic traits, blood pressure) and *vice versa* using bi-directional two-sample Mendelian randomization (MR) analysis.

**Methods:** We used 185 genetic variants associated with schizophrenia from the latest Psychiatric Genomics Consortium GWAS (*n* = 130,644) in the forward analysis (schizophrenia to cardiometabolic traits) and genetic variants associated with the cardiometabolic traits from various consortia in the reverse analysis (cardiometabolic traits to schizophrenia), both at genome-wide significance (5 × 10^−8^). The primary method was inverse-variance weighted MR, supported by supplementary methods such as MR-Egger, as well as median and mode-based methods.

**Results:** In the forward analysis, schizophrenia was associated with slightly higher low-density lipoprotein (LDL) cholesterol levels (0.013 SD change in LDL per log odds increase in schizophrenia risk, 95% CI, 0.001–0.024 SD; *p* = 0.027) and total cholesterol levels (0.013 SD change in total cholesterol per log odds increase in schizophrenia risk, 95% CI, 0.002–0.025 SD; *p* = 0.023). However, these associations did not survive multiple testing corrections. There was no evidence of a causal effect of cardiometabolic traits on schizophrenia in the reverse analysis.

**Discussion:** Dyslipidemia and obesity in schizophrenia patients are unlikely to be driven primarily by schizophrenia itself. Therefore, lifestyle, diet, antipsychotic drugs side effects, as well as shared mechanisms for metabolic dysfunction and schizophrenia such as low-grade systemic inflammation could be possible reasons for the apparent increased risk of metabolic disease in people with schizophrenia. Further research is needed to examine the shared immune mechanism hypothesis.

## 1 Introduction

Schizophrenia is a psychiatric disorder affecting approximately 1% of the world population (Goh et al., 2022). Compared to the general population, individuals with schizophrenia have significant reductions in average life expectancy by 14.5 years; this premature mortality is primarily attributed to physical illness, including type 2 diabetes mellitus and cardiovascular disease (Papanastasiou, 2013; Afzal et al., 2021; Goh et al., 2022). In addition, a systematic review and meta-analysis by Afzal et al. (2021) of 120 studies from 43 countries demonstrated that people with severe mental illness have a drastically higher prevalence and odds of obesity than the general population.

The reasons for adverse cardiovascular and metabolic health conditions within this patient population are complex and multifactorial. Despite well-documented cardiometabolic side effects of certain antipsychotic drugs, schizophrenia may have an independent effect, and this has been suggested by the high prevalence of metabolic syndrome in drug-naïve patients with schizophrenia (Papanastasiou, 2013; Smith et al., 2020; Goh et al., 2022). Indeed, antipsychotic-naïve patients with first-episode psychosis have a 2.5-fold risk for metabolic syndrome compared to age–and gender-matched controls (Goh et al., 2022). Furthermore, drug-naïve patients with schizophrenia and their unaffected first-degree relatives demonstrate several features of metabolic syndrome, such as increased visceral fat, dyslipidaemia, impaired glucose tolerance, and insulin resistance (Papanastasiou, 2013; Perry et al., 2016; Pillinger et al., 2017; Goh et al., 2022). Thus, antipsychotic medication is not the sole contributor to adverse cardiometabolic outcomes and schizophrenia itself may be a risk factor for the onset of metabolic syndrome.

The direction of this relationship is yet to be established. A longitudinal study demonstrated that persistently high fasting insulin levels from 9 years of age was associated with a higher risk of developing psychosis at 24 years, indicating possible early-life origins of the observed schizophrenia-diabetes association (Perry et al., 2021a). Individuals at clinical high risk for psychosis (i.e., do not have a diagnosis and are untreated) have shown metabolic abnormalities, such as dyslipidaemia, hypertension, obesity/overweight, and insulin resistance, which are not explained by medication adverse effects (Cadenhead et al., 2019; Dickens et al., 2021).

However, limitations of existing studies include possible confounding and reverse causation, which makes inferring causality difficult (Davies et al., 2018). We used a genetic epidemiological method called Mendelian randomization (MR) to examine whether schizophrenia is potentially causally related to cardiometabolic traits and *vice versa*. MR uses genetic variants as instrumental variables (IVs) to examine whether an exposure is likely to be causally related to an outcome (Davies et al., 2018). Genetic variants are randomly allocated during conception and are, therefore, independent of potential confounding environmental factors. However, for MR analysis, genetic variants are subject to three assumptions in order to be valid IVs and these assumptions must be evaluated when interpreting the results: they must be robustly associated with the exposure; they must not be associated with confounders; they must not affect the outcome unless it is through the exposure (i.e., pleiotropy is absent) (Davies et al., 2018; Teumer, 2018).

Previous MR studies have yielded discordant findings, and mostly focus on the cardiometabolic traits-schizophrenia relationship, with limited insight into potential the reverse association (Hartwig et al., 2016; Li et al., 2018; Polimanti et al., 2018; Adams et al., 2021; Aoki et al., 2022). Thus, in this study, a bidirectional, two-sample MR analysis was conducted using the largest summary-level dataset on schizophrenia from the Psychiatric Genomic Consortium (PGC), investigating the effect of schizophrenia on the risk of cardiometabolic traits, as well the effect of cardiometabolic traits on the risk of schizophrenia.

## 2 Materials and methods

### 2.1 Study design overview

**FIGURE 1:**
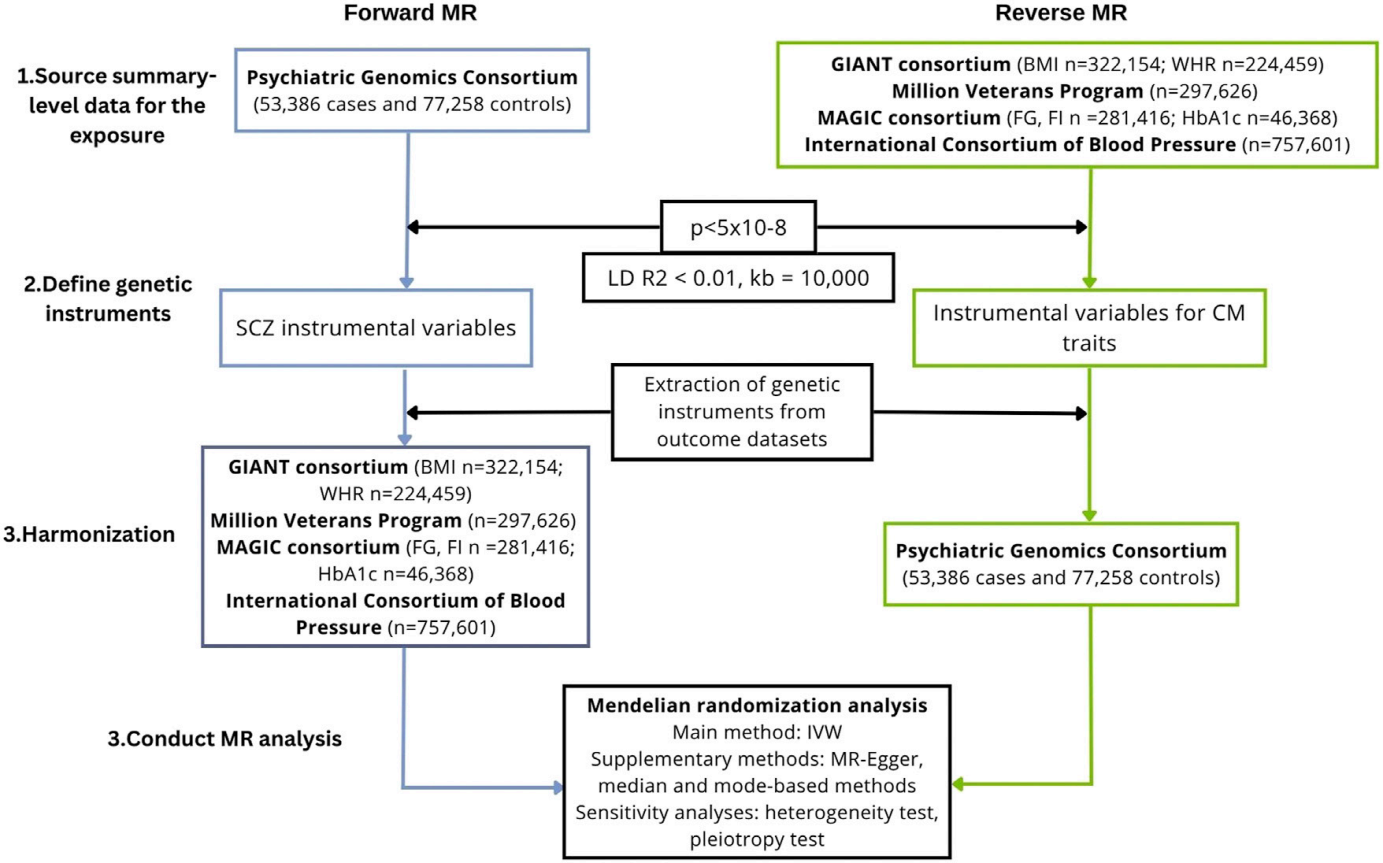
Study workflow of the two-sample, bidirectional MR analysis investigating the association between schizophrenia and cardiometabolic traits. BMI, body mass index; CM, cardiometabolic; GIANT, Genetic Investigation of ANthropometric Traits; IVW, inverse-variance weighted; FG, fasting glucose; FI, fasting insulin; LD, linkage disequilibrium; MAGIC, Meta-Analyses of Glucose and Insulin-related traits Consortium; MR, Mendelian randomization; SCZ, schizophrenia; SNPs, single-nucleotide polymorphisms; WHR, waist-hip ratio.

We conducted a bidirectional MR study to investigate the causal association of schizophrenia on cardiometabolic traits, including anthropometric traits [body mass index (BMI), waist-hip ratio (WHR)], glycaemic traits (HbA1c, fasting glucose, fasting insulin), blood lipids [triglycerides, high-density lipoprotein (HDL), low-density lipoprotein (LDL), total cholesterol] and blood pressure (systolic and diastolic blood pressure)*.* We also performed the analysis in the reverse direction, i.e., we investigated the causal association of cardiometabolic traits on schizophrenia. A flowchart presenting the study design is shown in Figure 1.

### 2.2 Data

To derive a reliable conclusion on the causal association between schizophrenia and cardiometabolic factors, a two-sample framework was used, i.e., the exposure and the outcome were measured using two non-overlapping samples. Summary-level datasets were obtained from large consortia of genome-wide association studies as summarized data are available for larger sample sizes, improving the power to detect a causal effect (Burgess et al., 2020). Due to the paucity of diverse datasets for some of the key traits, only studies with data on individuals of a European ancestry were included. Individual-level studies and multi-ancestry studies were excluded (unless they provided separate data for Europeans). The datasets used are summarized in Table 1.

**TABLE 1:** Sample characteristics for exposures and outcomes in the Mendelian randomization analysis. BMI, body mass index; GIANT, Genetic Investigation of Anthropometric Traits; MAGIC, Meta-Analyses of Glucose and Insulin-related traits Consortium; MVP, Million Veteran Program; PGC, Psychiatric Genomics Consortium; SD, standard deviation; WHR, waist-hip ratio.

Trait	Sample size	References	Consortium	Population	Units
Schizophrenia	53,386 cases and 77,258 controls	Trubetskoy et al. (2022)	PGC	European	Log odds
BMI	322,154	Locke et al. (2015)	GIANT	European	SD (kg/m^2^)
WHR	21,244	Shungin et al. (2015)	GIANT	European	SD
Blood lipids	215,551	Klarin et al. (2018)	MVP	European	SD (mg/dl)
Fasting glucose	200,622	Chen et al. (2021b)	MAGIC	European	mmol/l
Fasting insulin	151,013	Chen et al. (2021b)	MAGIC	European	pmol/l
Hba1c	46,368	Soranzo et al. (2010)	MAGIC	European	%
Systolic blood pressure	757,601	Evangelou et al. (2018b)	International Consortium of Blood Pressure	European	mmHg
Diastolic blood pressure	757,601	Evangelou et al. (2018b)	International Consortium of Blood Pressure	European	mmHg

The largest and most up-to-date GWAS was selected for schizophrenia from the PGC, including a total of 53,386 cases and 77,258 controls of European ancestry (Trubetskoy et al., 2022). The GWAS summary statistics were downloaded from the PGC website (available at https://www.med.unc.edu/pgc/). Cases were defined as individuals diagnosed with schizophrenia spectrum disorder based on DSM-IV criteria.

Summary-level data for BMI and WHR was selected from the Genetic Investigation of ANthropometric Traits (GIANT) consortium, including up to 322,154 and 21,244 individuals, respectively (Locke et al., 2015; Shungin et al., 2015) (available at https://portals.broadinstitute.org/collaboration/giant/index.php/GIANT_consortium). Summary data for blood lipids were obtained from the Million Veteran Program GWAS, including 215,551 individuals of European ancestry (Klarin et al., 2018). This data is available through dbGaP at https://www.ncbi.nlm.nih.gov/gap/using the accession number phs001672.v1.p1. For glycaemic traits, the MAGIC consortium was used (https://magicinvestigators.org/). Data for fasting glucose and fasting insulin were derived from a sample of 281,416 individuals, and HbA1c was derived from a sample of 46,368 individuals. Both samples included adults of European descent (Soranzo et al., 2010; Chen et al., 2021a). Summary-level data for blood pressure traits were selected from the United Kingdom Biobank and the International Consortium of Blood Pressure, including up to 757,601 individuals (Evangelou et al., 2018a). Summary statistics for blood pressure are available from the GWAS Catalog (https://www.ebi.ac.uk/gwas/publications/30224653).

### 2.3 Genetic instruments

To ensure that the genetic variants used in the analysis were valid IVs, several quality control steps were conducted using the TwoSampleMR package in R (Hemani et al., 2018). Firstly, the MR assumptions indicate that the IVs must be strongly associated with the exposure, thus, the SNPs were filtered and only SNPs strongly associated with the exposure at genome-wide significance (P < 5 × 10^−8^) were selected. Secondly, to determine linkage disequilibrium (LD) between SNPs, we used the clump_data function (Hemani et al., 2018). This function utilises the PLINK clumping method: SNPs in linkage disequilibrium 10,000 kb pairs apart at an *R* ^2^ threshold of 0.01 were pruned against the European 1000 Genomes reference panel. Among pairs of SNPs with *R* ^2^ above this threshold, the SNP with the strongest evidence of association with the key trait (smallest *p*-value) was retained and the other SNP in the pair was excluded. Genetic variants not found in the reference panel were excluded. Genetic variants not found in the reference panel were excluded. Finally, harmonization was conducted using the harmonise_data function as the MR analysis involved the use of two independent datasets with genetic variants which may not share the same allele pair. Thus, harmonization ensured that the effect of a SNP on the exposure, and the effect of the same SNP on the outcome, corresponded to the same allele (Burgess et al., 2020). Genetic variants that did not share the same allele pair between datasets were identified and corrected. Alternatively, palindromic SNPs, i.e., SNPs with alleles on the forward strand that are the same as on the reverse strand, were excluded from the analysis (Burgess et al., 2020). The SNPs that remained after this selection process were used as IVs in the MR analysis. Summary data of the genetic instruments were subsequently extracted from the outcome dataset, including effect of the SNP on the outcome (beta or odds ratio), standard error, *p*-value, effect allele, other allele, effect allele frequency, and sample size.

### 2.4 Statistical analyses

All statistical analyses were conducted using the TwoSampleMR package in R. Statistical significance was defined as *p* < 0.05. Individual SNP estimates (β_IV_) were obtained using the ratio method, where the effect of the SNP on the outcome (β_ZY_) was divided by the corresponding effect of the SNP from the exposure (β_ZX_) (Teumer, 2018).

$$\beta_{IV}=\beta_{ZY}/\beta_{ZX}$$

The ratio estimates were subsequently pooled using inverse variance weighted (IVW) analysis to derive an IVW effect estimate (Teumer, 2018). However, the IVW method requires that all SNPs are valid instruments (there is no horizontal pleiotropy) or are invalid in a way that the overall bias is zero (the horizontal pleiotropy is balanced). Thus, IVW analysis was followed by weighted median method, which allows up to 50% of the SNPs to be invalid instruments, i.e., violate the MR assumption, providing unbiased effect estimates even in the presence of unbalanced horizontal pleiotropy. In addition, the weighted mode clusters the IVs based on the similarity of their estimates, and the cluster with the greatest number of SNPs is chosen and is given the most weight for as the final causal estimate. If the IVs contributing to the largest cluster are unbiased, then the causal estimate from this method is unbiased (Hemani et al., 2018).

Altogether, we tested 11 different causal associations using univariable MR analysis. To account for multiple testing, we used a Bonferroni-corrected *p*-value of *p* < 0.05/11 = 5 × 10^−3^ as being statistically significant. A *p*-value <0.05 was suggestive evidence of a causal association.

### 2.5 Sensitivity analyses

Sensitivity analyses was conducted using the TwoSampleMR package in R. MR-Egger regression was conducted to test for pleiotropy. If the horizontal pleiotropic effects are in a particular direction, constraining the slope to go through zero will lead to bias. Thus, MR-Egger allows the intercept to pass through a value other than zero rather than constraining the slope to go through zero. This method, therefore, returns an unbiased estimate even if the IVs are invalid (Hemani et al., 2018). Heterogeneity between the estimates was quantified using Cochran’s Q statistic using the IVW method and MR-Egger regression. Finally, a “leave-one-out” analysis was performed whereby the MR was repeated while sequentially excluding each SNP to identify any SNPs with a potentially large effect.

## 3 Results

### 3.1 Potential causal effect of schizophrenia on cardiometabolic traits

In the forward analysis, up to 185 LD-independent SNPs significantly associated with schizophrenia were identified (Supplementary Table S1). However, not all these SNPs were found in the summary-level dataset for the cardiometabolic traits. In addition, palindromic SNPs were excluded in the harmonization process. This left 164, 164, 163, 153, 117, 80, 150, 150, 178, 178, and 93 SNPs as IVs for MR analyses of schizophrenia on HDL, LDL, triglycerides, total cholesterol, BMI, WHR, systolic blood pressure, diastolic blood pressure, fasting glucose, fasting insulin and HbA1c, respectively. IVW effect estimates were computed for each cardiometabolic trait in turn. This was followed by computing effect estimates using additional robust methods (MR-Egger, weighted-median and -mode) to address instrumental validity. Cochran’s Q statistic was calculated to quantify heterogeneity between estimates and a “leave-one-out” analysis was conducted to identify any SNPs with a potentially large effect.

**FIGURE 2:**
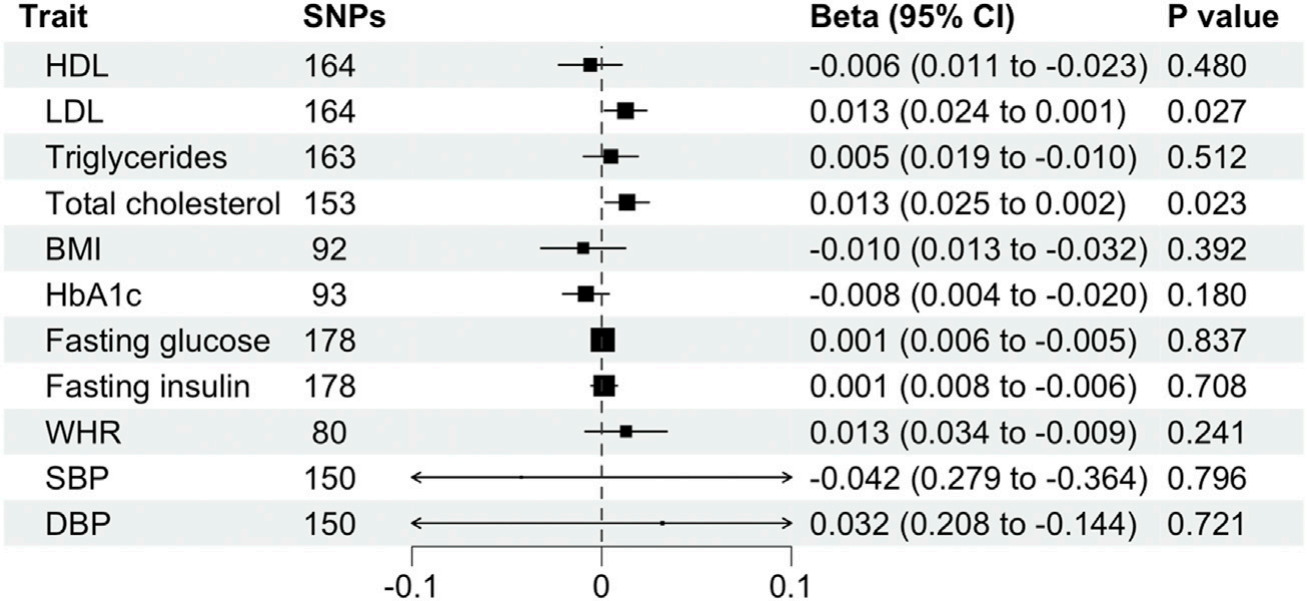
Mendelian randomization estimates (beta and 95% confidence intervals) for the association between schizophrenia (exposure) and cardiometabolic traits (outcome) using the inverse variance weighted method. BMI, body mass index; CI, confidence interval; DBP, diastolic blood pressure; HDL, high-density lipoprotein; LDL, low-density lipoprotein; SBP, systolic blood pressure; SNP, single nucleotide polymorphism; TC, total cholesterol; WHR, waist-hip ratio.

**FIGURE 3:**
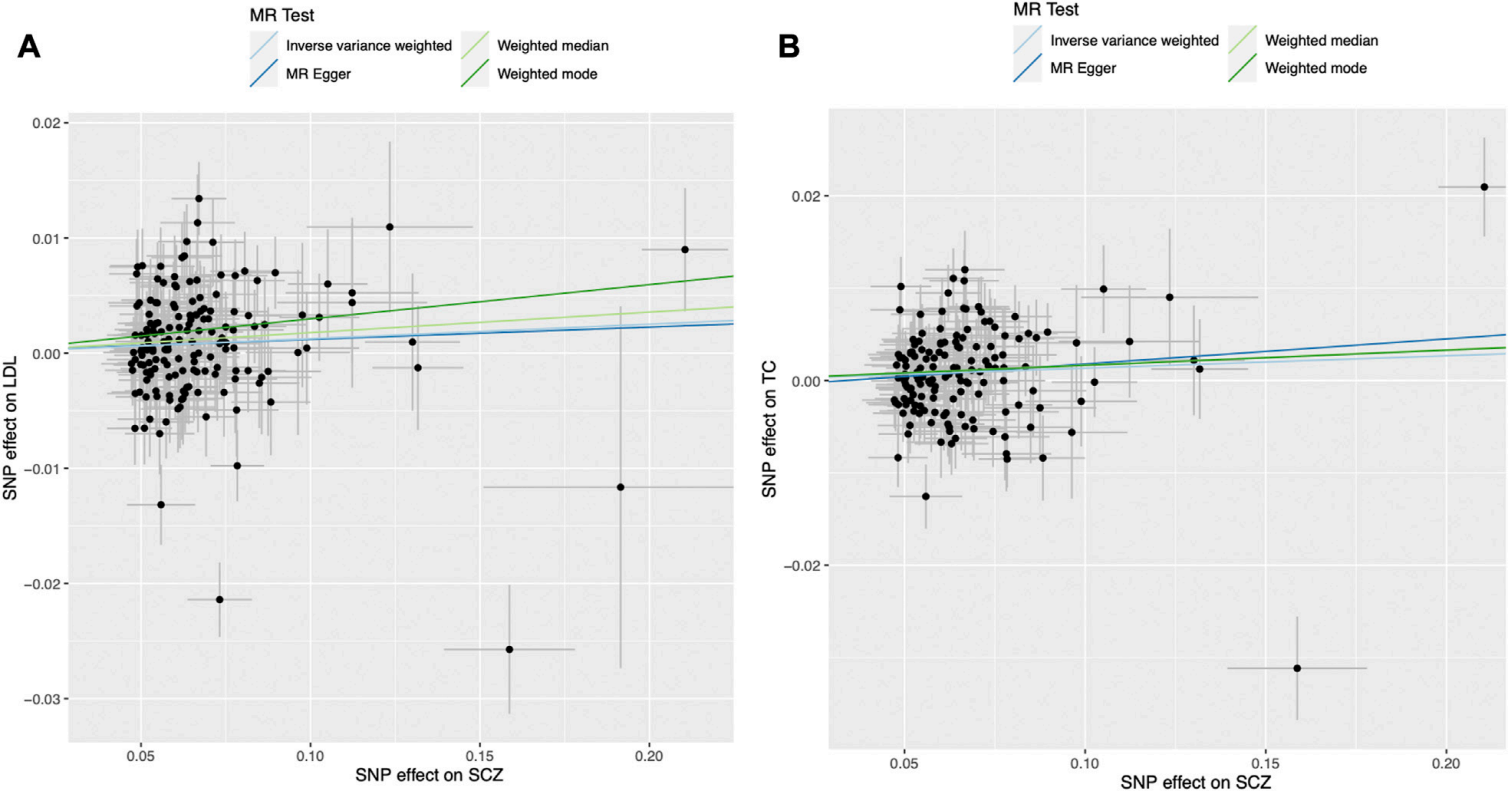
Mendelian randomization scatter plot for the association between schizophrenia (exposure) and **(A)** LDL and **(B)** total cholesterol (outcomes). Each black dot represents the estimate of an individual genetic variant and its corresponding 95% confidence interval. LDL, low-density lipoprotein; MR, Mendelian randomisation; SCZ, schizophrenia; SNP, single nucleotide polymorphism.

We found evidence for associations between schizophrenia and LDL (0.013 SD change in LDL per log odds increase in schizophrenia, 95% CI, 0.001–0.024 SD; *p* = 0.027) and total cholesterol level (0.013 SD change in total cholesterol per log odds increase in schizophrenia, 95% CI, 0.002–0.025 SD; *p* = 0.023) using the primary IVW analysis method (Figure 2). The effect sizes for the causal association between schizophrenia and LDL and total cholesterol were relatively consistent across the different methods (Supplementary Table S2). This is further demonstrated in their respective scatter plots (Figure 3). However, the effect sizes for these associations are very small and did not survive correction for multiple testing. Using the MR-Egger regression test, we did not find evidence for horizontal pleiotropy for LDL or total cholesterol. The MR-Egger intercept provided no evidence against the null hypothesis of no unmeasured pleiotropy (LDL, intercept *p* = 0.937; total cholesterol, intercept *p* = 0.563). Iterative removal of each individual SNP using leave-one-out analysis did not affect the IVW estimates for LDL or TC, suggesting that they were not driven by one singular SNP (Supplementary Figure S1E,F). However, Cochran’s Q statistic demonstrated evidence of heterogeneity between the effect estimates between the 164 LDL and 153 total cholesterol associated genetic variants (LDL, heterogeneity *p* = 8.80 × 10-10; total cholesterol, heterogeneity *p* = 1.11 × 10-9).

Furthermore, schizophrenia was not associated with BMI (β, −0.010 SD; 95% CI, −0.032–0.013 SD; *p* = 0.392), WHR (β, 0.013 SD; 95%, CI −0.009–0.034 SD; *p* = 0.241), HDL (β, −0.006 SD; 95% CI, −0.023–0.011 SD; *p* = 0.480), triglycerides (β, 0.005 SD; 95% CI, −0.010–0.019 SD; *p* = 0.512), fasting glucose (β, 0.001 mmol/L; 95% CI, −0.005–0.006 mmol/L; *p* = 0.837), fasting insulin (β, 0.001 pmol/L; 95% CI, −0.006–0.008 pmol/L; *p* = 0.708), HbA1c (β, −0.008%; 95% CI, −0.020%–0.004%; *p* = 0.180), systolic blood pressure (β, −0.042 mmHg; 95% CI, −0.364–0.279 mmHg; *p* = 0.796) or diastolic blood pressure (β, 0.032 mmHg; 95% CI, −0.144–0.208 mmHg; *p* = 0.721) using the primary IVW analysis method (Figure 2) or other methods (Supplementary Table S2). The MR-Egger intercept revealed generally minimal pleiotropy and leave-one-out analysis demonstrated robustness of the effect estimates (Supplementary Table S2, Supplementary Figure S1). However, Cochran’s Q test demonstrated heterogeneity for all traits except HbA1c (Supplementary Table S2).

### 3.2 Potential causal effect of cardiometabolic traits on schizophrenia

In the reverse analysis, 105, 71, 93, 72, 68, 29, 455, 454, 87, 43, and 11 LD-independent, genome-wide significant SNPs were identified for HDL, LDL, triglycerides, total cholesterol, BMI, WHR, systolic blood pressure, diastolic blood pressure, fasting glucose, fasting insulin and HbA1c, and respectively. After excluding SNPs missing in the summary-level dataset for schizophrenia and palindromic SNPs, 101, 65, 84, 66, 67, 28, 392, 393, 75, 38, and 11 SNPs remained as instrumental variables. Effect estimates were derived using IVW, MR-Egger, weighted-median and -mode analysis. Finally, sensitivity analyses were conducted: Cochran’s Q statistic and “leave-one-out” analysis.

**FIGURE 4:**
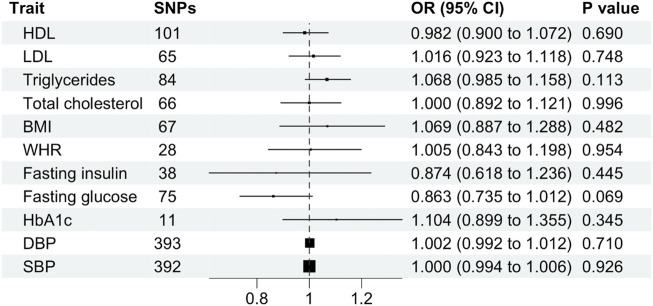
Mendelian randomization estimates (odds ratio and 95% confidence intervals) for the association between cardiometabolic traits (exposure) and schizophrenia (outcome) using the inverse variance weighted method. BMI, body mass index; CI, confidence interval; DBP, diastolic blood pressure; HDL, high-density lipoprotein; LDL, low-density lipoprotein; OR, odds ratio; SBP, systolic blood pressure; SNP, single nucleotide polymorphism; WHR, waist-hip ratio.

Cardiometabolic traits were not associated with schizophrenia, including BMI (OR, 1.069; 95% CI, 0.887–1.288; *p* = 0.482), WHR (OR, 1.005; 95% CI 0.843–1.198 SD; *p* = 0.954), HDL (OR, 0.982; 95% CI, 0.900–1.072; *p* = 0.690), LDL (OR, 1.016; 95% CI, 0.923–1.118; *p* = 0.512), total cholesterol (OR, 1.000; 95% CI, 0.892–1.121; *p* = 0.996), triglycerides (OR, 1.068; 95% CI, 0.985–1.158; *p* = 0.113), fasting glucose (OR, 0.863; 95% CI, 0.735–1.012; *p* = 0.069), fasting insulin (OR, 0.874; 95% CI, 0.618–1.236; *p* = 0.445), HbA1c (OR, 1.104; 95% CI, 0.899–1.355; *p* = 0.345), systolic blood pressure (OR, 1.000; 95% CI, 0.994–1.006; *p* = 0.926) or diastolic blood pressure (OR, 1.002; 95% CI, 0.992–1.012; *p* = 0.710) and schizophrenia using the primary IVW analysis method (Figure 4) or other methods (Supplementary Table S3). Leave-one-out analysis demonstrated robustness of the effect estimates (Supplementary Figure S2). However, the MR-Egger intercept indicated potential pleiotropy for LDL and HbA1c. In addition, Cochran’s Q test demonstrated heterogeneity for all traits except HbA1c (Supplementary Table S3).

## 4 Discussion

In this study, we conducted bidirectional two-sample MR analyses using publicly available large-scale genomic summary data to examine potential causal effects of schizophrenia on cardiometabolic traits and *vice versa*. Our data do not suggest evidence for a possible causal effect of schizophrenia on cardiometabolic traits, or of cardiometabolic traits on schizophrenia. Although we report a potential causal effect of schizophrenia on LDL and total cholesterol levels, the magnitude of these associations were small and did not survive multiple testing correction. Taken together, our findings suggest that cardiometabolic alteration in schizophrenia patients is unlikely to be fully attributable to an independent effect of schizophrenia on these outcomes. Rather, dyslipidaemia and obesity in schizophrenia patients may be attributable to other factors such as lifestyle, adverse effects of antipsychotic medications, as well as inflammation.

Individuals with schizophrenia show deficits in cognition, perception, and volition, which can impact their activities of daily living, self-care, finances, and lifestyle (Henderson et al., 2015). For example, they are more likely to have low physical activity, a diet with high-calorie fast foods (also related to income) and higher rates of alcohol and tobacco consumption (Henderson et al., 2015; Goh et al., 2022). Evidence shows that healthcare services struggle to engage people with severe mental illness in screening and other health-promoting campaigns and that tailored interventions are required to reach this population successfully (Osborn et al., 2019; Hassan et al., 2020).

The self-medication hypothesis proposes that patients with schizophrenia may use substances to cope with their symptoms (Awad and Voruganti, 2015). In the United States, up to 75% of individuals with schizophrenia are smokers, compared to 25% of the general population. A similar Spanish study found that 54.5% of patients with schizophrenia in Spain are current daily smokers, compared to 31.5% of the general population. Smokers were also significantly more likely to be affected by a cardiovascular event than the non-smokers (Henderson et al., 2015). An MR study by Wootton et al. (2020) supported this hypothesis by demonstrating that genetic liability for schizophrenia was significantly associated with lifetime smoking. However, evidence was stronger for smoking as a risk factor for schizophrenia, indicating a potential bidirectional mechanism.

Aside from the aforementioned risk factors, the use of second-generation antipsychotics by patients with schizophrenia have been shown to lead to key features of metabolic syndrome, including weight gain, obesity, impaired glucose tolerance, and dyslipidaemia (Henderson et al., 2015; Goh et al., 2022; Richards-Belle et al., 2023). Despite their benefits to treat symptoms of psychosis, clozapine and olanzapine are most commonly linked to these cardiometabolic traits (Huhn et al., 2019). Indeed, 52% of patients undergoing clozapine treatment demonstrate metabolic syndrome (Mitchell et al., 2013). These traits may be attributed to the effect of antipsychotics on the hypothalamus, which activates hunger and inhibits satiety, which subsequently affects lipid and glucose metabolism by acting on the liver, pancreatic β-cells, adipose tissue, and skeletal muscle in the periphery. For example, adiponectin is a cytokine secreted by the adipose tissue with insulin-sensitising and anti-inflammatory effects (Achari and Jain, 2017). Patients with schizophrenia treated with antipsychotics demonstrate lower adiponectin levels, particularly those with metabolic syndrome, compared with healthy controls. These patients had increased insulin resistance, hypertension, hypertriglyceridemia, and lower HDL levels. Leptin is also an adipokine involved in regulating energy balance by inhibiting hunger. Previous studies have shown that patients with schizophrenia taking antipsychotics have higher leptin levels, particularly in those taking second-generation antipsychotics (Chen et al., 2020; Goh et al., 2022). In a recent study by Zhang et al. (2021) leptin levels of patients with schizophrenia were positively correlated with BMI. Thus, the use of antipsychotic medication may initiate a vicious cycle whereby increased adipose tissue mass induces a state of hyperleptinaemia, increasing appetite suppression to regulate energy balance. However, hyperleptinaemia leads to a lack of sensitivity to leptin, also known as leptin resistance, ultimately contributing to an increased appetite, further weight gain and further leptin production (Chen et al., 2021b; Genchi et al., 2021; Goh et al., 2022).

Alternatively, previous studies have demonstrated that inflammation may play an important role in mediating this association. Perry et al. (2021b) conducted an MR study and identified a weak association between HDL, triglycerides and schziophrenia, which increased in strength when using inflammation-related IVs. Similarly, MR studies have demonstrated a causal link between inflammatory markers and schizophrenia risk (Lin et al., 2019; Perry et al., 2021b; Khandaker et al., 2021; Williams et al., 2022). Thus, inflammation may be a common cause for cardiometabolic traits and schizophrenia.

In this study, we employed a bidirectional MR framework, which avoided reverse causality and minimized residual confounding. We built upon previous MR studies using an updated set of instruments of schizophrenia from the PGC, thus improving the power to detect a causal association and accurately estimate the magnitude of the effect. We also included a complete set of traits (blood lipids, anthropometric traits, blood pressure, and glycaemic traits) to be comprehensive and fully representative of metabolic syndrome. However, genetic variants are subject to three assumptions which must be considered when interpreting the results. The first assumption, which indicates that the genetic variants are associated with the exposure of interest, was satisfied by excluding SNPs that did not reach genome-wide significance (P > 5 × 10^−8^). The second assumption which states that the genetic variants must not be associated with confounders should be satisfied as the genetic variants are randomly allocated during conception. The third assumption, which requires the genetic variants do not affect the outcome unless it is through the exposure, is difficult to explicitly test but our sensitivity analyses indicated that pleiotropy was unlikely to affect the results. Furthermore, this study was restricted to individuals of European ancestry as these were the datasets with appropriate sizes to enable the MR analysis. Nevertheless, despite using the largest dataset available for schizophrenia, our study could still have lacked statistical power. Whether these results also apply to other populations will require investigating in diverse, large-scale samples which are currently being collected.

In conclusion, using a bidirectional MR framework we found that the relationship between schizophrenia and various cardiometabolic traits is unlikely to be a causal one. Thus, multiple hypotheses to account for this relationship has been raised in the literature, including lifestyle factors (e.g., smoking, diet, activity), antipsychotic medication, inflammation, among others. Ultimately, we need further research with larger global populations to elucidate the links between schizophrenia and metabolic syndrome.

## Data Availability

The original contributions presented in the study are included in the article/Supplementary Material, further inquiries can be directed to the corresponding authors.
