# Supplementary material for: Schizophrenia and cardiometabolic abnormalities: A Mendelian randomization study
Source: Front Genet. 2023 Apr 6;14:1150458. doi: 10.3389/fgene.2023.1150458 (PMC10115959; doi:10.3389/fgene.2023.1150458)
Supplement: Supplementary file 1 [file DataSheet1.docx]

Supplementary Material

Schizophrenia and Cardiometabolic Abnormalities: A Mendelian Randomization Study

**Noushin Saadullah Khani^1^*, Marius Cotic^1,2^, Baihan Wang^1^, Rosemary Abidoph^1,3^, Georgina Mills^1^, Alvin Richards-Belle^1,4^, Benjamin I Perry^5,6^, Golam M Khandaker^7,8,9^, and Elvira Bramon^1,3^**

^1^ Mental Health Neuroscience Department, Division of Psychiatry, University College London, London, W1T 7BN, UK

^2^ Department of Genetics & Genomic Medicine, UCL Great Ormond Street Institute of Child Health, University College London, London, UK

^3^ Camden and Islington NHS Foundation Trust, 4 St Pancras Way, London, NW1 0PE, UK

^4^ Epidemiology and Applied Clinical Research Department, Division of Psychiatry, University College London, London W1T 7BN, UK

^5^ Department of Psychiatry, University of Cambridge, Cambridge, UK

^6^ Cambridgeshire and Peterborough NHS Foundation Trust, Cambridge, UK

^7^ MRC Integrative Epidemiology Unit, Population Health Sciences, Bristol Medical School, University of Bristol, Bristol, UK

^8^ NIHR Bristol Biomedical Research Centre, Bristol, UK

^9^ Avon and Wiltshire Mental Health Partnership NHS Trust, Bristol, UK

*** Correspondence:**Noushin Saadullah Khani and Elvira Bramon
[noushin.khani.21@ucl.ac.uk](mailto:noushin.khani.21@ucl.ac.uk)

e.bramon@ucl.ac.uk

# Supplementary Tables

**Supplementary Table 1.** Summary of the genetic variants used as instrumental variables in the Mendelian randomization analysis. SE, standard error; SNP, single nucleotide polymorphism.

| SNP | Effect allele | Other allele | P value | Beta | SE |
| --- | --- | --- | --- | --- | --- |
| rs2332700 | C | G | 3.88E-14 | 0.075 | 0.009 |
| rs167924 | A | G | 2.34E-08 | -0.050 | 0.009 |
| rs4575535 | A | G | 5.77E-09 | -0.056 | 0.010 |
| rs73292401 | T | A | 5.48E-10 | -0.068 | 0.012 |
| rs57433322 | C | G | 1.99E-09 | 0.083 | 0.013 |
| rs2455415 | C | T | 1.69E-08 | -0.049 | 0.009 |
| rs39967 | T | C | 4.38E-08 | -0.062 | 0.012 |
| rs2333321 | A | G | 1.25E-11 | 0.071 | 0.010 |
| rs61405217 | C | T | 7.03E-09 | 0.050 | 0.008 |
| rs7191183 | T | C | 3.32E-10 | -0.058 | 0.010 |
| rs61786047 | G | A | 8.34E-09 | 0.078 | 0.012 |
| rs6001259 | C | T | 3.70E-08 | -0.191 | 0.040 |
| rs11210892 | G | A | 2.68E-12 | 0.064 | 0.008 |
| rs1604060 | A | G | 3.24E-08 | -0.077 | 0.015 |
| rs35734242 | T | C | 1.37E-08 | -0.051 | 0.009 |
| rs9461856 | G | A | 5.71E-13 | -0.062 | 0.009 |
| rs2071277 | T | C | 2.64E-14 | 0.067 | 0.008 |
| rs7830315 | T | C | 3.08E-08 | -0.048 | 0.009 |
| rs6984242 | G | A | 3.86E-10 | 0.055 | 0.008 |
| rs60135207 | G | T | 1.53E-08 | 0.050 | 0.008 |
| rs187557 | C | T | 2.03E-08 | 0.067 | 0.011 |
| rs1901512 | T | C | 5.72E-10 | 0.058 | 0.009 |
| rs61857878 | A | T | 4.44E-09 | 0.060 | 0.010 |
| rs2909457 | G | A | 1.48E-08 | 0.049 | 0.008 |
| rs113264400 | T | C | 2.87E-08 | -0.112 | 0.022 |
| rs331395 | C | G | 5.55E-09 | -0.061 | 0.011 |
| rs1914399 | C | G | 1.40E-08 | 0.049 | 0.008 |
| rs4702 | G | A | 2.79E-21 | 0.084 | 0.008 |
| rs2710323 | T | C | 1.23E-19 | 0.078 | 0.008 |
| rs7647398 | C | T | 1.07E-12 | 0.077 | 0.010 |
| rs6482437 | A | C | 3.33E-12 | -0.099 | 0.015 |
| rs3770754 | C | G | 5.35E-09 | 0.053 | 0.009 |
| rs12303743 | G | C | 1.59E-09 | -0.087 | 0.016 |
| rs2456020 | C | T | 1.13E-15 | 0.082 | 0.009 |
| rs56205728 | G | A | 1.01E-10 | -0.063 | 0.010 |
| rs2255663 | C | T | 8.40E-10 | 0.058 | 0.009 |
| rs13107325 | C | T | 2.90E-21 | -0.159 | 0.019 |
| rs4766428 | C | T | 3.93E-17 | -0.075 | 0.010 |
| rs16851048 | T | C | 4.15E-12 | -0.074 | 0.011 |
| rs308697 | C | A | 8.83E-09 | 0.050 | 0.008 |
| rs12877581 | G | C | 1.80E-09 | -0.060 | 0.010 |
| rs9318627 | A | C | 4.35E-12 | 0.061 | 0.008 |
| rs17194490 | G | T | 1.80E-11 | -0.078 | 0.012 |
| rs12712510 | T | C | 5.14E-11 | 0.057 | 0.008 |
| rs17571951 | T | C | 9.97E-10 | -0.064 | 0.011 |
| rs12883788 | C | T | 1.86E-12 | -0.061 | 0.009 |
| rs778371 | A | G | 1.50E-17 | -0.081 | 0.010 |
| rs13011472 | C | G | 4.28E-16 | -0.070 | 0.009 |
| rs4779050 | T | G | 7.27E-11 | 0.058 | 0.008 |
| rs11638554 | T | G | 7.58E-12 | 0.065 | 0.009 |
| rs11680723 | C | G | 2.05E-14 | -0.086 | 0.012 |
| rs4812325 | G | A | 8.96E-16 | -0.072 | 0.009 |
| rs145071536 | T | C | 1.62E-12 | -0.085 | 0.013 |
| rs11090045 | G | A | 5.12E-09 | -0.056 | 0.010 |
| rs5995756 | T | C | 3.18E-11 | 0.057 | 0.008 |
| rs5751191 | T | C | 3.00E-14 | -0.066 | 0.009 |
| rs500102 | T | C | 4.87E-09 | 0.052 | 0.008 |
| rs113113059 | T | C | 4.89E-08 | 0.058 | 0.010 |
| rs2078266 | A | G | 2.94E-08 | 0.070 | 0.012 |
| rs9304548 | C | A | 1.59E-08 | 0.057 | 0.009 |
| rs6673880 | A | G | 7.20E-12 | -0.062 | 0.010 |
| rs3795310 | C | T | 5.75E-09 | 0.051 | 0.008 |
| rs3791710 | T | C | 3.02E-08 | 0.060 | 0.010 |
| rs1384292 | G | C | 3.05E-08 | -0.049 | 0.009 |
| rs634940 | G | T | 1.78E-11 | -0.066 | 0.010 |
| rs6925964 | A | T | 3.11E-08 | 0.098 | 0.016 |
| rs72974238 | C | A | 8.74E-09 | 0.053 | 0.009 |
| rs4636654 | G | A | 4.89E-08 | 0.048 | 0.008 |
| rs11027839 | A | C | 2.40E-09 | -0.052 | 0.009 |
| rs6798742 | A | G | 4.57E-11 | -0.061 | 0.010 |
| rs741896 | C | G | 2.17E-09 | -0.054 | 0.010 |
| rs3824451 | T | C | 2.54E-08 | -0.066 | 0.012 |
| rs6546857 | A | G | 2.74E-09 | -0.060 | 0.011 |
| rs17016552 | C | G | 1.20E-08 | 0.052 | 0.009 |
| rs10861176 | G | A | 1.59E-08 | -0.056 | 0.010 |
| rs16825349 | A | G | 7.72E-10 | -0.069 | 0.012 |
| rs13016542 | T | C | 8.28E-12 | 0.088 | 0.012 |
| rs708228 | C | T | 6.56E-09 | -0.053 | 0.010 |
| rs11664298 | G | A | 8.94E-13 | -0.077 | 0.012 |
| rs76838079 | C | T | 1.53E-08 | -0.078 | 0.015 |
| rs498591 | A | T | 2.11E-09 | -0.072 | 0.013 |
| rs35351411 | A | C | 2.21E-13 | -0.064 | 0.009 |
| rs7515363 | C | T | 1.84E-09 | 0.054 | 0.008 |
| rs10108980 | C | T | 2.73E-09 | -0.063 | 0.011 |
| rs11136325 | G | A | 3.05E-09 | 0.054 | 0.009 |
| rs4129585 | A | C | 5.11E-18 | 0.075 | 0.008 |
| rs79445414 | T | C | 2.80E-08 | -0.123 | 0.025 |
| rs7001340 | T | C | 3.17E-08 | 0.058 | 0.010 |
| rs35531336 | A | G | 2.22E-08 | 0.074 | 0.012 |
| rs728055 | T | A | 8.85E-14 | 0.067 | 0.008 |
| rs7801375 | A | G | 7.56E-10 | -0.073 | 0.013 |
| rs77502336 | G | C | 1.25E-08 | -0.053 | 0.010 |
| rs12293670 | A | G | 1.56E-14 | 0.070 | 0.009 |
| rs6125656 | G | A | 6.29E-09 | -0.064 | 0.012 |
| rs926288 | A | G | 2.50E-08 | -0.061 | 0.012 |
| rs12138231 | T | A | 7.99E-09 | -0.067 | 0.012 |
| rs58120505 | T | C | 2.24E-24 | 0.090 | 0.008 |
| rs72943392 | G | C | 2.39E-08 | -0.053 | 0.010 |
| rs12969453 | A | G | 8.52E-14 | 0.065 | 0.008 |
| rs1789595 | A | T | 4.04E-11 | 0.064 | 0.009 |
| rs4632195 | C | T | 4.59E-08 | -0.047 | 0.009 |
| rs9636107 | A | G | 5.12E-16 | -0.070 | 0.009 |
| rs6974218 | A | C | 6.80E-10 | 0.055 | 0.008 |
| rs7803571 | C | T | 1.27E-12 | 0.064 | 0.008 |
| rs2514218 | C | T | 1.35E-14 | 0.070 | 0.009 |
| rs12771371 | G | A | 1.94E-08 | 0.052 | 0.009 |
| rs1540840 | G | C | 2.21E-09 | 0.056 | 0.009 |
| rs9876421 | C | T | 9.19E-12 | -0.063 | 0.010 |
| rs6549963 | T | C | 4.31E-08 | 0.048 | 0.008 |
| rs10873538 | T | G | 3.01E-13 | -0.067 | 0.010 |
| rs10117 | G | A | 4.66E-10 | 0.055 | 0.008 |
| rs9687282 | T | G | 7.33E-09 | -0.053 | 0.010 |
| rs1430894 | C | T | 6.15E-10 | -0.053 | 0.009 |
| rs12489270 | T | C | 7.47E-11 | -0.058 | 0.009 |
| rs699318 | T | C | 2.27E-13 | 0.067 | 0.009 |
| rs1451488 | A | G | 4.47E-16 | -0.071 | 0.009 |
| rs11993663 | C | A | 3.32E-08 | -0.050 | 0.009 |
| rs4921741 | A | G | 1.21E-08 | -0.056 | 0.010 |
| rs1427633 | G | C | 4.10E-08 | 0.048 | 0.008 |
| rs2238057 | T | G | 8.50E-22 | -0.084 | 0.009 |
| rs3814883 | C | T | 1.58E-14 | 0.067 | 0.008 |
| rs215412 | G | A | 2.69E-10 | -0.058 | 0.010 |
| rs11693094 | C | T | 4.29E-10 | 0.054 | 0.008 |
| rs62183855 | A | C | 2.66E-09 | 0.066 | 0.010 |
| rs12129573 | C | A | 2.28E-18 | -0.078 | 0.010 |
| rs4700418 | C | G | 5.37E-16 | -0.070 | 0.009 |
| rs505061 | C | A | 5.80E-10 | -0.053 | 0.009 |
| rs7575796 | A | G | 2.07E-08 | 0.096 | 0.015 |
| rs12310367 | A | G | 1.21E-08 | -0.052 | 0.009 |
| rs1615350 | C | T | 4.92E-14 | 0.074 | 0.009 |
| rs79210963 | T | C | 4.14E-10 | -0.086 | 0.015 |
| rs8055219 | G | A | 5.69E-11 | -0.067 | 0.011 |
| rs1198588 | A | T | 1.73E-21 | -0.103 | 0.012 |
| rs6701322 | A | G | 6.15E-12 | -0.069 | 0.011 |
| rs1000237 | T | A | 2.80E-16 | -0.073 | 0.009 |
| rs72986630 | C | T | 3.59E-10 | -0.112 | 0.020 |
| rs56335113 | A | G | 6.02E-12 | 0.065 | 0.009 |
| rs11263861 | G | A | 2.02E-08 | -0.052 | 0.010 |
| rs246024 | C | T | 3.61E-08 | 0.048 | 0.008 |
| rs117799466 | G | C | 1.28E-10 | -0.062 | 0.010 |
| rs7900775 | T | C | 3.82E-08 | 0.049 | 0.008 |
| rs11191580 | T | C | 1.77E-17 | 0.132 | 0.013 |
| rs79780963 | C | T | 3.39E-17 | 0.131 | 0.013 |
| rs6715366 | G | A | 2.49E-08 | -0.054 | 0.010 |
| rs12285419 | C | A | 1.05E-14 | -0.085 | 0.012 |
| rs17731 | G | A | 4.37E-09 | -0.052 | 0.009 |
| rs2381411 | T | C | 1.25E-08 | -0.050 | 0.009 |
| rs6943762 | T | C | 1.57E-15 | 0.105 | 0.012 |
| rs13233308 | C | T | 1.75E-08 | 0.049 | 0.008 |
| rs10086619 | A | G | 4.97E-10 | -0.072 | 0.012 |
| rs9454727 | A | G | 3.35E-08 | 0.054 | 0.009 |
| rs2815731 | C | A | 4.39E-11 | 0.060 | 0.008 |
| rs217336 | C | A | 8.05E-09 | 0.050 | 0.008 |
| rs6538539 | G | T | 4.43E-11 | 0.057 | 0.008 |
| rs10777956 | A | G | 1.75E-08 | -0.050 | 0.009 |
| rs10894308 | G | A | 8.18E-10 | 0.054 | 0.008 |
| rs7115692 | G | A | 6.32E-10 | -0.062 | 0.010 |
| rs4936215 | A | G | 1.87E-14 | 0.082 | 0.010 |
| rs893949 | C | T | 1.64E-12 | 0.061 | 0.008 |
| rs3016386 | G | A | 6.24E-09 | 0.050 | 0.008 |
| rs2252074 | T | G | 6.19E-15 | -0.069 | 0.009 |
| rs10876446 | G | C | 1.03E-08 | -0.054 | 0.010 |
| rs61937595 | C | T | 1.15E-15 | 0.130 | 0.014 |
| rs1881046 | G | T | 3.39E-08 | 0.051 | 0.009 |
| rs13195636 | A | C | 6.55E-40 | 0.211 | 0.013 |
| rs1915019 | A | G | 6.57E-09 | 0.057 | 0.009 |
| rs16867571 | A | G | 2.68E-10 | 0.066 | 0.010 |
| rs4672366 | A | T | 2.80E-08 | 0.054 | 0.009 |
| rs2053079 | A | G | 3.01E-09 | -0.060 | 0.011 |
| rs2532240 | C | T | 2.58E-11 | 0.061 | 0.008 |
| rs55938136 | A | G | 1.23E-08 | 0.061 | 0.010 |
| rs2999392 | C | T | 3.05E-08 | -0.052 | 0.010 |
| rs11941714 | G | A | 3.07E-08 | 0.052 | 0.009 |
| rs7634476 | A | G | 5.46E-11 | -0.058 | 0.009 |
| rs1892346 | T | A | 3.56E-08 | -0.048 | 0.009 |
| rs11807834 | G | A | 2.98E-08 | -0.055 | 0.010 |
| rs11587347 | C | G | 1.53E-12 | -0.104 | 0.016 |
| rs149165 | T | G | 3.01E-08 | 0.048 | 0.008 |
| rs10035564 | A | G | 4.38E-13 | -0.067 | 0.010 |
| rs713692 | G | A | 2.67E-09 | -0.057 | 0.010 |
| rs73229090 | C | A | 4.34E-13 | 0.103 | 0.013 |
| rs7251 | C | G | 8.29E-12 | 0.064 | 0.009 |
| rs11740474 | A | T | 1.13E-09 | -0.054 | 0.009 |
| rs72802868 | G | T | 4.55E-13 | 0.069 | 0.009 |
| rs12652777 | T | C | 1.52E-08 | 0.049 | 0.008 |

**Supplementary Table 2.** Mendelian randomization estimates (beta and standard error) for the association between schizophrenia (exposure) and cardiometabolic traits (outcome) using the inverse variance weighted method, MR-egger and weighted median- and mode-based methods. BMI, body mass index; HDL, high-density lipoprotein; IVW, inverse variance-weighted; LDL, low-density lipoprotein; MR, Mendelian randomization; SE, standard error; nSNP, number of single nucleotide polymorphisms used in the analysis.

| Trait | Cochran’s Q P value | MR-Egger intercept P value | nSNP | Method | β (SE) | P |
| --- | --- | --- | --- | --- | --- | --- |
| HDL | 4.68x10^-59^ | 0.085 | 164 | IVW | -0.006 (0.008) | 0.480 |
|  |  |  | 164 | MR-Egger | -0.065 (0.035) | 0.066 |
|  |  |  | 164 | Weighted median | -0.009 (0.007) | 0.237 |
|  |  |  | 164 | Weighted mode | -0.022 (0.022) | 0.315 |
| LDL | 1.19x10^-9^ | 0.937 | 164 | IVW | 0.013 (0.006) | 0.027 |
|  |  |  | 164 | MR-Egger | 0.011 (0.024) | 0.649 |
|  |  |  | 164 | Weighted median | 0.018 (0.007) | 0.006 |
|  |  |  | 164 | Weighted mode | 0.030 (0.016) | 0.065 |
| Triglycerides | 3.70x10^-33^ | 0.037 | 163 | IVW | 0.005 (0.006) | 0.512 |
|  |  |  | 163 | MR-Egger | 0.067 (0.024) | 0.029 |
|  |  |  | 163 | Weighted median | 0.001 (0.007) | 0.882 |
|  |  |  | 163 | Weighted mode | -0.007 (0.022) | 0.727 |
| Total cholesterol | 1.109x10^-9^ | 0.563 | 153 | IVW | 0.013 (0.006) | 0.023 |
|  |  |  | 153 | MR-Egger | 0.027 (0.024) | 0.267 |
|  |  |  | 153 | Weighted median | 0.017 (0.007) | 0.013 |
|  |  |  | 153 | Weighted mode | 0.016 (0.022) | 0.454 |
| BMI | 5.00x10^-20^ | 0.921 | 117 | IVW | -0.010 (0.011) | 0.392 |
|  |  |  | 117 | MR-Egger | -0.014 (0.043) | 0.749 |
|  |  |  | 117 | Weighted median | 0.005 (0.012) | 0.688 |
|  |  |  | 117 | Weighted mode | 0.012 (0.023) | 0.612 |
| Waist-to-hip ratio | 4.86x10^-7^ | 0.646 | 80 | IVW | 0.013 (0.011) | 0.241 |
|  |  |  | 80 | MR-Egger | -0.006 (0.041) | 0.894 |
|  |  |  | 80 | Weighted median | 0.013 (0.013) | 0.325 |
|  |  |  | 80 | Weighted mode | 0.015 (0.026) | 0.558 |
| Systolic blood pressure | 0.00 | 0.484 | 150 | IVW | -0.042 (0.164) | 0.796 |
|  |  |  | 150 | MR-Egger | 0.404 (0.658) | 0.540 |
|  |  |  | 150 | Weighted median | 0.052 (0.093) | 0.572 |
|  |  |  | 150 | Weighted mode | 0.240 (0.247) | 0.333 |
| Diastolic blood pressure | 8.86x10^-317^ | 0.208 | 150 | IVW | 0.032 (0.090) | 0.721 |
|  |  |  | 150 | MR-Egger | 0.469 (0.357) | 0.191 |
|  |  |  | 150 | Weighted median | -0.039 (0.050) | 0.438 |
|  |  |  | 150 | Weighted mode | -0.210 (0.180) | 0.247 |
| Fasting glucose | 1.36x10^-3^ | 0.131 | 178 | IVW | -0.148 (0.081) | 0.069 |
|  |  |  | 178 | MR-Egger | -0.184 (0.155) | 0.239 |
|  |  |  | 178 | Weighted median | -0.126 (0.092) | 0.171 |
|  |  |  | 178 | Weighted mode | -0.157 (0.080) | 0.053 |
| Fasting insulin | 4.89x10^-8^ | 0.149 | 178 | IVW | -0.135 (0.177) | 0.445 |
|  |  |  | 178 | MR-Egger | -0.046 (0.536) | 0.932 |
|  |  |  | 178 | Weighted median | -0.029 (0.156) | 0.852 |
|  |  |  | 178 | Weighted mode | -0.017 (0.216) | 0.936 |
| HbA1c | 0.691 | 0.629 | 93 | IVW | -0.008 (0.006) | 0.180 |
|  |  |  | 93 | MR-Egger | -0.019 (0.023) | 0.407 |
|  |  |  | 93 | Weighted median | -0.009 (0.009) | 0.311 |
|  |  |  | 93 | Weighted mode | -0.012 (0.017) | 0.479 |

**Supplementary Table 3.** Mendelian randomization estimates (beta and standard error) for the association between cardiometabolic traits (exposure) and schizophrenia (outcome) using the inverse variance weighted method, MR-egger and weighted median- and mode-based methods. BMI, body mass index; HDL, high-density lipoprotein; IVW, inverse variance-weighted; LDL, low-density lipoprotein; MR, Mendelian randomization; SE, standard error; SNP, single nucleotide polymorphism; nSNP, number of single nucleotide polymorphisms used in the analysis.

| Trait | Cochran’s Q P value | MR-Egger intercept P value | nSNP | Method | β (SE) | P |
| --- | --- | --- | --- | --- | --- | --- |
| HDL | 1.11x10^-31^ | 0.306 | 101 | IVW | -0.018 (0.045) | 0.690 |
|  |  |  | 101 | MR-Egger | -0.078 (0.073) | 0.292 |
|  |  |  | 101 | Weighted median | -0.041 (0.041) | 0.308 |
|  |  |  | 101 | Weighted mode | -0.052 (0.038) | 0.168 |
| LDL | 9.39x10^-11^ | 0.015 | 65 | IVW | 0.016 (0.049) | 0.748 |
|  |  |  | 65 | MR-Egger | -0.149 (0.081) | 0.070 |
|  |  |  | 65 | Weighted median | -0.050 (0.049) | 0.306 |
|  |  |  | 65 | Weighted mode | -0.052 (0.047) | 0.276 |
| Triglycerides | 1.21x10^-16^ | 0.164 | 84 | IVW | 0.065 (0.041) | 0.113 |
|  |  |  | 84 | MR-Egger | 0.003 (0.061) | 0.964 |
|  |  |  | 84 | Weighted median | 0.048 (0.038) | 0.209 |
|  |  |  | 84 | Weighted mode | 0.058 (0.033) | 0.084 |
| Total cholesterol | 1.16x10^-19^ | 0.537 | 66 | IVW | 0.000 (0.058) | 0.023 |
|  |  |  | 66 | MR-Egger | -0.058 (0.110) | 0.267 |
|  |  |  | 66 | Weighted median | 0.050 (0.052) | 0.013 |
|  |  |  | 66 | Weighted mode | 0.024 (0.051) | 0.454 |
| BMI | 6.01x10^-33^ | 0.022 | 67 | IVW | 0.067 (0.095) | 0.482 |
|  |  |  | 67 | MR-Egger | 0.665 (0.272) | 0.017 |
|  |  |  | 67 | Weighted median | 0.074 (0.085) | 0.385 |
|  |  |  | 67 | Weighted mode | 0.168 (0.185) | 0.368 |
| Waist-to-hip ratio | 0.026 | 0.606 | 28 | IVW | 0.005 (0.090) | 0.954 |
|  |  |  | 28 | MR-Egger | -0.207 (0.416) | 0.623 |
|  |  |  | 28 | Weighted median | -0.036 (0.116) | 0.758 |
|  |  |  | 28 | Weighted mode | -0.094 (0.158) | 0.554 |
| Systolic blood pressure | 4.36x10^-101^ | 0.090 | 392 | IVW | 0.000 (0.003) | 0.926 |
|  |  |  | 392 | MR-Egger | 0.013 (0.008) | 0.109 |
|  |  |  | 392 | Weighted median | 0.003 (0.003) | 0.354 |
|  |  |  | 392 | Weighted mode | 0.004 (0.006) | 0.539 |
| Diastolic blood pressure | 3.74x10^-99^ | 0.294 | 393 | IVW | 0.002 (0.005) | 0.710 |
|  |  |  | 393 | MR-Egger | 0.014 (0.013) | 0.267 |
|  |  |  | 393 | Weighted median | 0.004 (0.005) | 0.510 |
|  |  |  | 393 | Weighted mode | 0.006 (0.011) | 0.551 |
| Fasting glucose | 3.93x10^-9^ | 0.783 | 75 | IVW | 0.001 (0.003) | 0.837 |
|  |  |  | 75 | MR-Egger | -0.016 (0.012) | 0.156 |
|  |  |  | 75 | Weighted median | 0.003 (0.004) | 0.480 |
|  |  |  | 75 | Weighted mode | 0.012 (0.012) | 0.353 |
| Fasting insulin | 2.68x10^-9^ | 0.861 | 38 | IVW | 0.001 (0.004) | 0.708 |
|  |  |  | 38 | MR-Egger | 0.022 (0.015) | 0.136 |
|  |  |  | 38 | Weighted median | -0.002 (0.004) | 0.723 |
|  |  |  | 38 | Weighted mode | -0.001 (0.011) | 0.918 |
| HbA1c | 0.127 | 0.009 | 11 | IVW | 0.099 (0.105) | 0.345 |
|  |  |  | 11 | MR-Egger | 0.434 (0.225) | 0.085 |
|  |  |  | 11 | Weighted median | 0.110 (0.124) | 0.378 |
|  |  |  | 11 | Weighted mode | 0.386 (0.144) | 0.023 |

**Supplementary Figure 1.** Leave-one-out analysis for schizophrenia on various cardiometabolic traits: (a) schizophrenia on BMI (b) schizophrenia on WHR (c) schizophrenia on triglycerides (d) schizophrenia on HDL (e) schizophrenia on LDL (f) schizophrenia on total cholesterol (g) schizophrenia on HbA1c (h) schizophrenia on fasting insulin (i) schizophrenia on fasting glucose (j) schizophrenia on diastolic blood pressure (k) schizophrenia on systolic blood pressure. Circles represent the IVW estimate for schizophrenia on the cardiometabolic outcomes and horizontal bars indicate 95% confidence intervals. BMI, body mass index; DBP, diastolic blood pressure; FG, fasting glucose; FI, fasting insulin; HDL, high-density lipoprotein; LDL, low-density lipoprotein; MR, Mendelian randomization; SBP, systolic blood pressure; TC, total cholesterol; TG, triglycerides; WHR, waist-hip ratio.

| **(a)** | **(b)** |
| --- | --- |
|  |  |
|  |  |
|  |  |
|  |  |
|  |  |
|  |  |
|  |  |
|  |  |
|  |  |
|  |  |
|  |  |
|  |  |
| **(c)** | **(d)** |
|  |  |
|  |  |
| **(e)** | **(f)** |
|  |  |
|  |  |
|  |  |
|  |  |
|  |  |
|  |  |
|  |  |
|  |  |
|  |  |
|  |  |
|  |  |
| **(g)** | **(h)** |
|  |  |
| **(i)** | **(j)** |
|  |  |
|  |  |
|  |  |
|  |  |
|  |  |
|  |  |
|  |  |
|  |  |
|  |  |
|  |  |
|  |  |
| **(k)** |  |
|  |  |

**Supplementary Figure 2.** Leave-one-out analysis for cardiometabolic traits on schizophrenia: (a) BMI on schizophrenia (b) WHR on schizophrenia (c) triglycerides on schizophrenia (d) HDL on schizophrenia (e) LDL on schizophrenia (f) total cholesterol on schizophrenia (g) HbA1c on schizophrenia (h) fasting insulin on schizophrenia (i) fasting glucose on schizophrenia (j) diastolic blood pressure on schizophrenia (k) systolic blood pressure on schizophrenia. Circles represent the IVW estimate for cardiometabolic outcomes on schizophrenia and horizontal bars indicate 95% confidence intervals. BMI, body mass index; DBP, diastolic blood pressure; FG, fasting glucose; FI, fasting insulin; HDL, high-density lipoprotein; LDL, low-density lipoprotein; MR, Mendelian randomization; SBP, systolic blood pressure; TC, total cholesterol; TG, triglycerides; WHR, waist-hip ratio.

| **(a)** | **(b)** |
| --- | --- |
|  |  |
| **(c)** | **(d)** |
|  |  |
|  |  |
|  |  |
| **(e)** | **(f)** |
|  |  |
|  |  |
|  |  |
|  |  |
|  |  |
|  |  |
|  |  |
|  |  |
| **(g)** | **(h)** |
|  |  |
| **(i)** | **(j)** |
|  |  |
|  |  |
|  |  |
|  |  |
|  |  |
|  |  |
|  |  |
|  |  |
|  |  |
|  |  |
|  |  |
| **(k)** |  |
|  |  |
